# 833. Efficacy and Safety of Long-Acting Cabotegravir + Rilpivirine in Participants with HIV/HCV Co-infection: ATLAS-2M 48-Week Results

**DOI:** 10.1093/ofid/ofab466.1029

**Published:** 2021-12-04

**Authors:** Ronald D’Amico, Paul Benn, Shanker Thiagarajah, Susan L Ford, Eileen Birmingham, Ojesh R Upadhyay, Louise Garside, Rodica Van Solingen-Ristea, Kati Vandermeulen, William Spreen

**Affiliations:** 1 ViiV Healthcare, Research Triangle Park, NC; 2 GlaxoSmithKline, London, England, United Kingdom; 3 Janssen Research and Development, Raritan, New Jersey; 4 Janssen Research & Development, LLC, Beerse, Antwerpen, Belgium; 5 Janssen R&D, Beerse, Antwerpen, Belgium

## Abstract

**Background:**

The phase IIIb ATLAS-2M study demonstrated non-inferiority of long-acting (LA) cabotegravir (CAB) + rilpivirine (RPV) dosed every 8 weeks (Q8W) compared with every 4 weeks (Q4W) for maintenance of virologic suppression. Hepatitis C virus (HCV) co-infection occurs in ~6% of people with HIV due to shared modes of transmission. We report efficacy and safety of CAB + RPV LA in participants with HIV/HCV co-infection in ATLAS-2M.

**Methods:**

Participants with HIV-1 RNA < 50 c/mL receiving CAB + RPV LA Q4W (transitioned from ATLAS [NCT02951052]) or oral comparator ART were randomized 1:1 to receive CAB + RPV LA Q4W or Q8W. Baseline HCV RNA was assessed by polymerase chain reaction. Participants with symptomatic chronic HCV infection requiring treatment within 12 months or liver enzymes not meeting entry criteria were excluded. Week 48 assessments included proportion with HIV-1 RNA ≥50 and < 50 c/mL (Snapshot algorithm), general and hepatic safety, and pharmacokinetics.

**Results:**

HIV/HCV co-infection was present in 10 (1%) of 1045 participants, 60% of whom were female at birth. At Week 48, 9/10 (90%) and 972/1035 (94%) participants with HIV/HCV co-infection and HIV mono-infection, respectively, had HIV-1 RNA < 50 c/mL (adjusted difference, 4.1; 95% CI, −14.5 to 22.6). No participants with HIV/HCV co-infection had HIV-1 RNA ≥50 c/mL (vs 14/1035 [1%] with HIV mono-infection) or confirmed virologic failure through Week 48 (vs 10 [1%] with HIV mono-infection); 1/10 (10%) discontinued for reasons other than adverse events (AEs). Excluding injection site reactions (ISRs), AEs and serious AEs were reported in 4 (40%) and 0 participants with HIV/HCV co-infection, respectively; the only AE reported in >1 participant was injection site pain (n=5; 50%). In participants with HIV/HCV co-infection, all ISRs were grade 1/2; none led to withdrawal. No hepatic laboratory abnormalities were reported in participants with HIV/HCV co-infection through Week 48; rates were low in those with HIV mono-infection (Table). Plasma CAB and RPV concentrations were similar between groups.

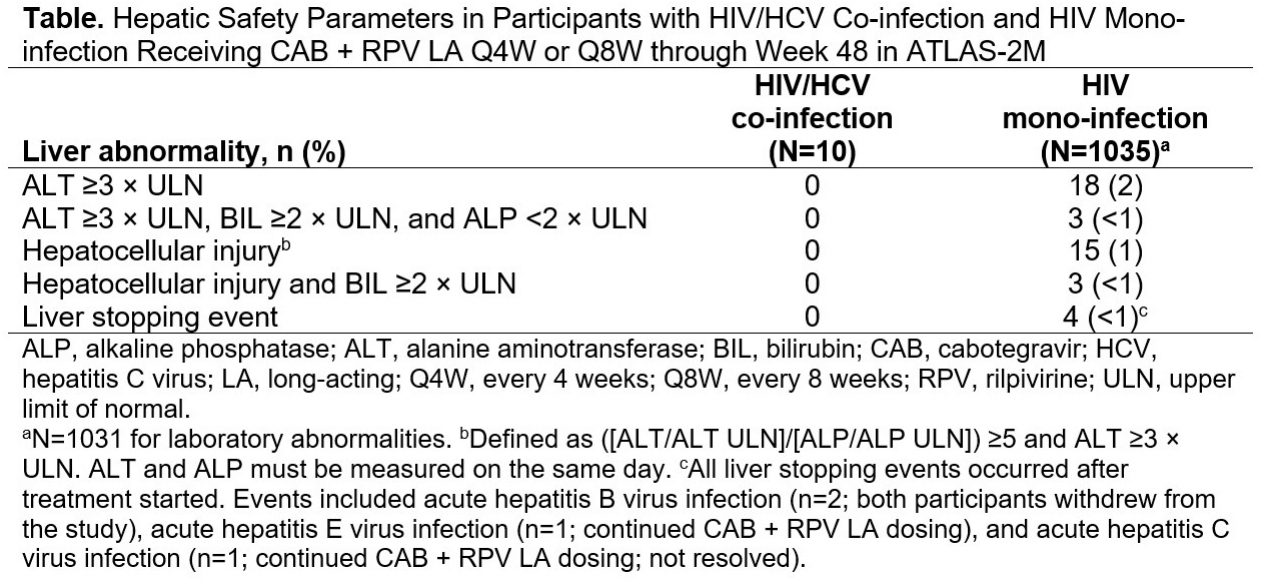

**Conclusion:**

CAB + RPV LA was effective and well tolerated in this small cohort of participants with HIV and asymptomatic HCV co-infection.

**Disclosures:**

**Ronald D’Amico, DO, MSc**, **GlaxoSmithKline** (Shareholder)**ViiV Healthcare** (Employee) **Paul Benn, MB ChB FRCP**, **ViiV Healthcare** (Employee) **Shanker Thiagarajah, MB ChB**, **GlaxoSmithKline** (Employee, Shareholder) **Susan L. Ford, PharmD**, **GlaxoSmithKline** (Shareholder)**ViiV Healthcare** (Employee) **Eileen Birmingham, MD, MPH**, **Janssen Research and Development** (Employee, Shareholder) **Ojesh R. Upadhyay, MPH, MBA**, **GlaxoSmithKline** (Employee) **Louise Garside, PhD**, **GlaxoSmithKline** (Employee) **Rodica Van Solingen-Ristea, MD**, **Janssen Research and Development** (Employee)**ViiV Healthcare** (Employee) **Kati Vandermeulen, M.SC.**, **Janssen Research and Development** (Employee) **William Spreen, PharmD**, **GlaxoSmithKline** (Shareholder)**ViiV Healthcare** (Employee)

